# Supplementary material for: Sustainable Preparation of Nanoporous Carbons via Dry Ball Milling: Electrochemical Studies Using Nanocarbon Composite Electrodes and a Deep Eutectic Solvent as Electrolyte
Source: Nanomaterials (Basel). 2021 Nov 30;11(12):3258. doi: 10.3390/nano11123258 (PMC8709160; doi:10.3390/nano11123258)
Supplement: Supplementary file 1 [file nanomaterials-11-03258-s001.zip › nanomaterials-1470539-supplementary.pdf]

nanomaterials

Supporting Information File

Sustainable Preparation of Nanoporous Carbons via Dry Ball Milling: Electrochemical Studies Using Nanocarbon Composite Electrodes and a Deep Eutectic Solvent as Electrolyte

Ana T. S. C. Brandão, Renata Costa, A. Fernando Silva and Carlos M. Pereira *

Departamento de Química e Bioquímica, Faculdade de Ciências da Universidade do Porto, CIQUP–Physical Analytical Chemistry and Electrochemistry Group, Rua do Campo Alegre, s/n, 4169–007 Porto, Portugal; up200706627@edu.fc.up.pt (A.T.S.C.B.); renata.costa@fc.up.pt (R.C.); afssilva@fc.up.pt (A.F.S.)

* Correspondence: cmpereir@fc.up.pt

Morphological studies: SEM analysis

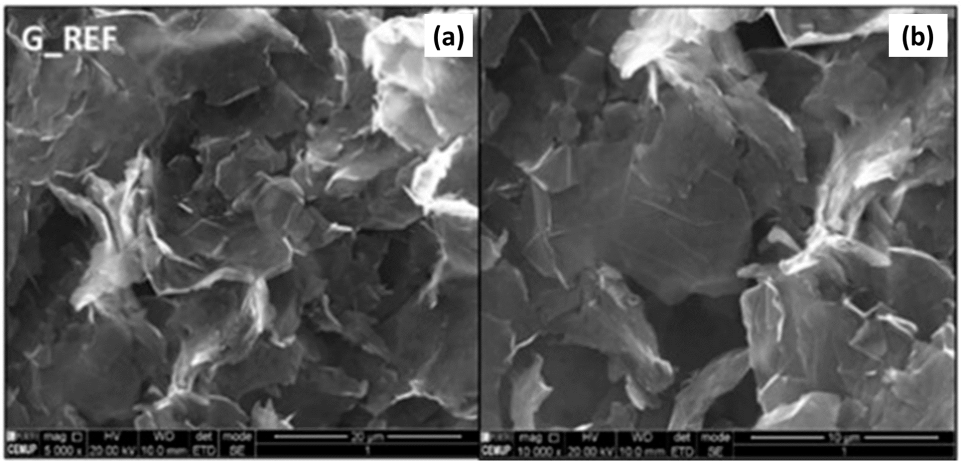

(A)

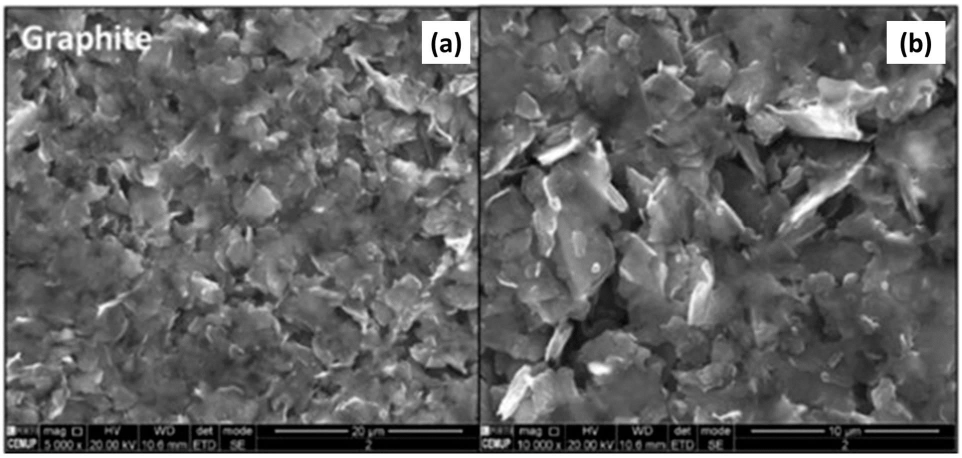

(B)

Figure S1. Electron microscopy images showing the structure of G_REF (**A**) and commercial graphite (**B**) samples with 5000× (a), 10000× (b) magnification.

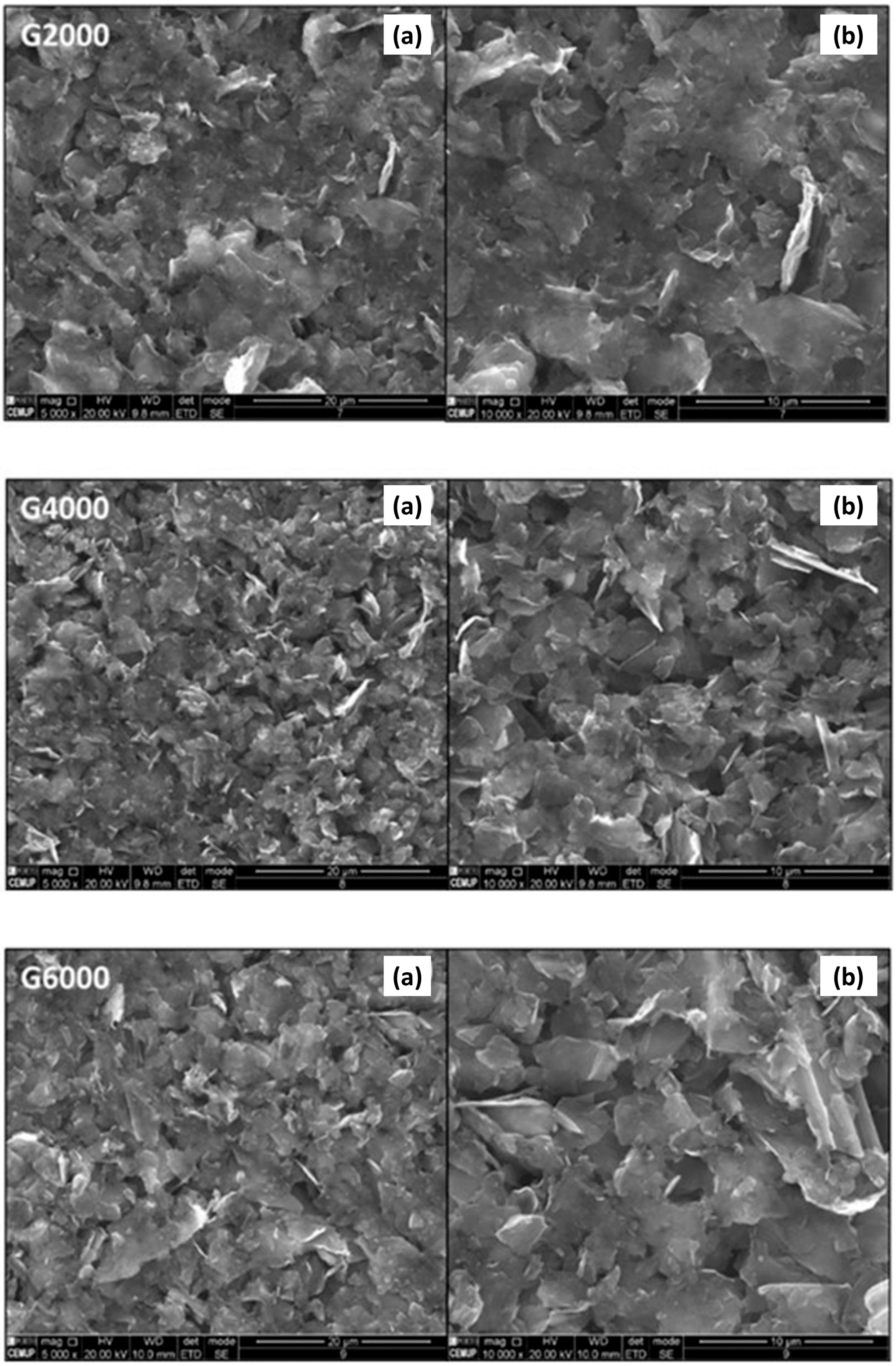

Figure S2. Electron microscopy images showing the structure of G@2000, G@4000 and G@6000 sample with 5000× (**a**), 10000× (**b**) magnification.

AFM analysis

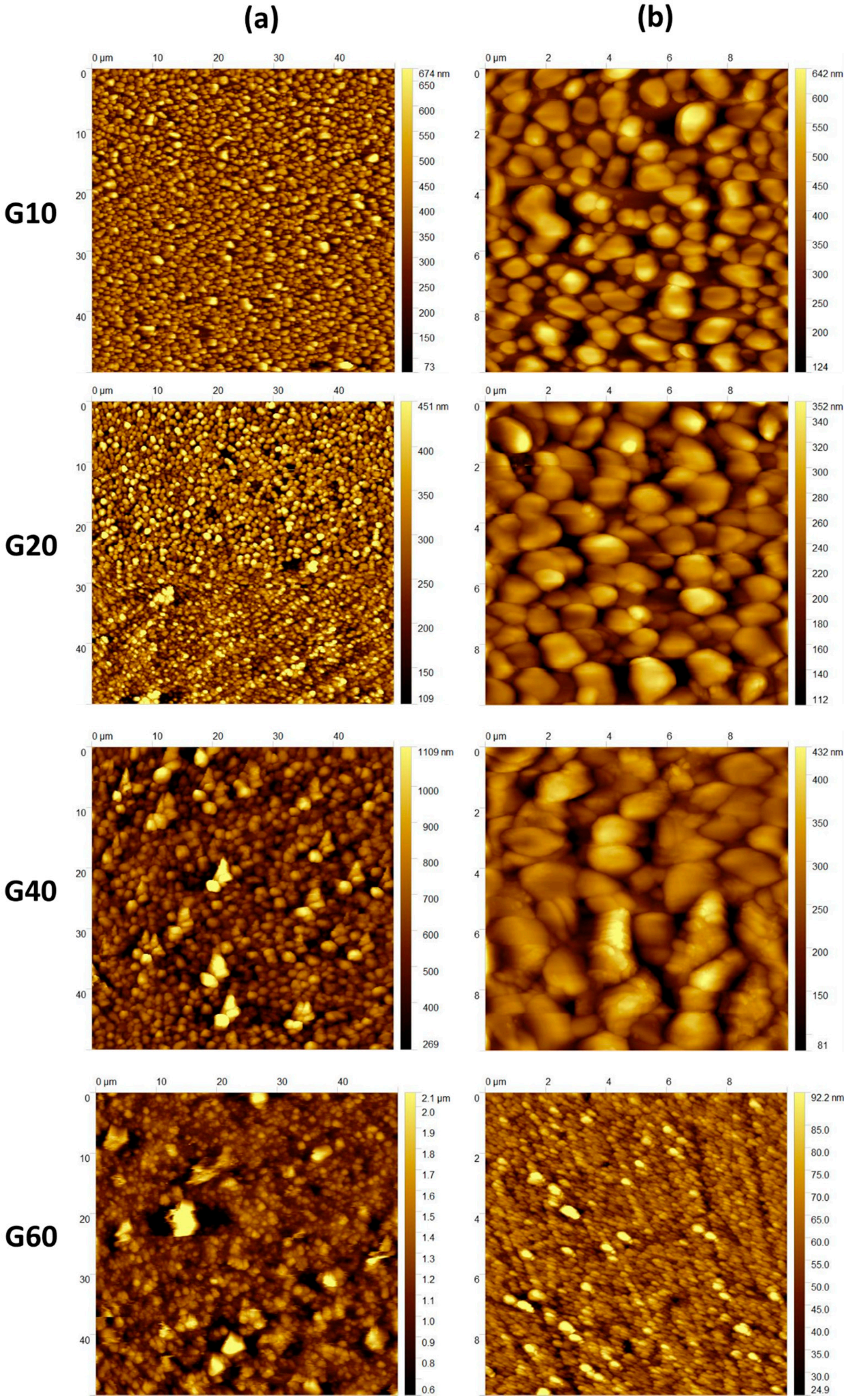

Figure S3. AFM topography images of samples G10 to G60 in GC substrate at 50 µm x 50 µm (**a**) and 10 µm × 10 µm (**b**) resolution.

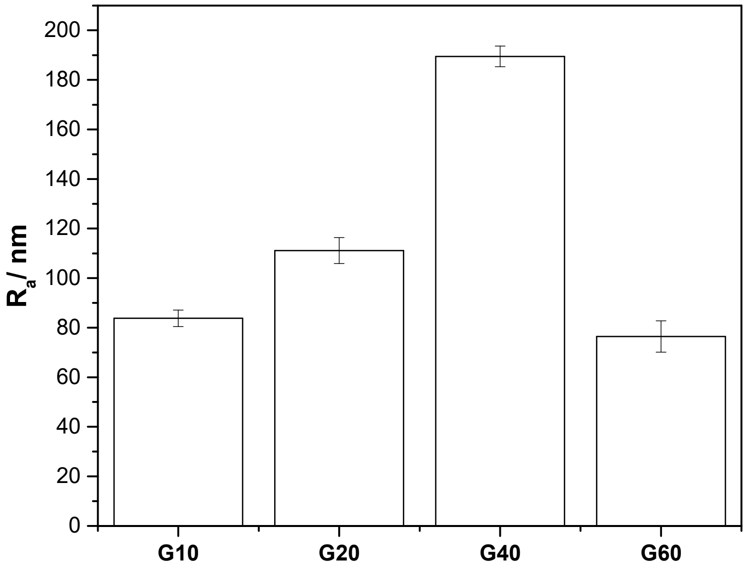

Figure S4. Surface roughness (Ra) obtained from the AFM images for Samples G10 to G60, at 50 μm × 50 μm resolution.

Raman Analysis

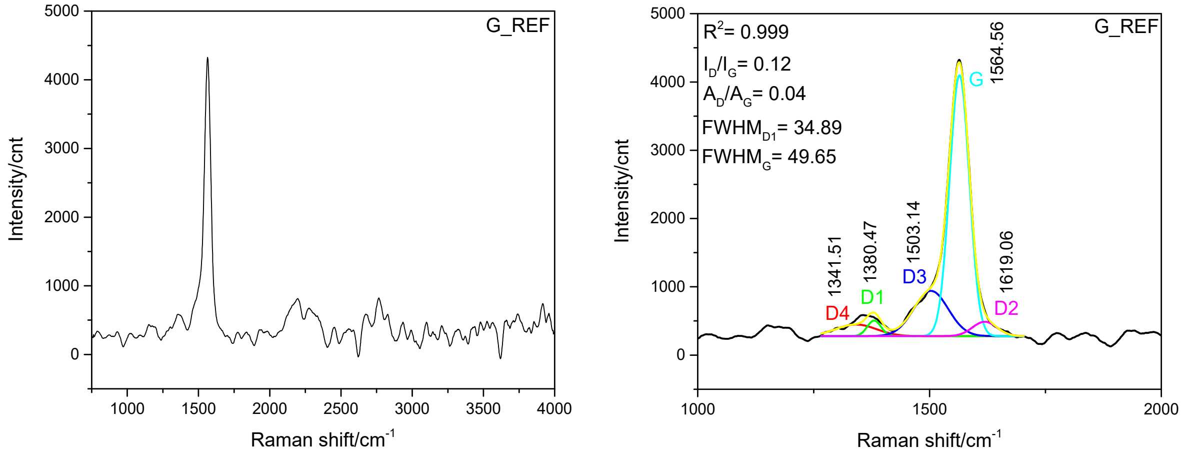

(a) (b)

Figure S5. Raman spectra of G_REF (**a**) and the deconvolution of the peaks from the 1st order Raman region of the signal emitted by G_REF (**b**). The sum of the deconvolution is marked with yellow line.

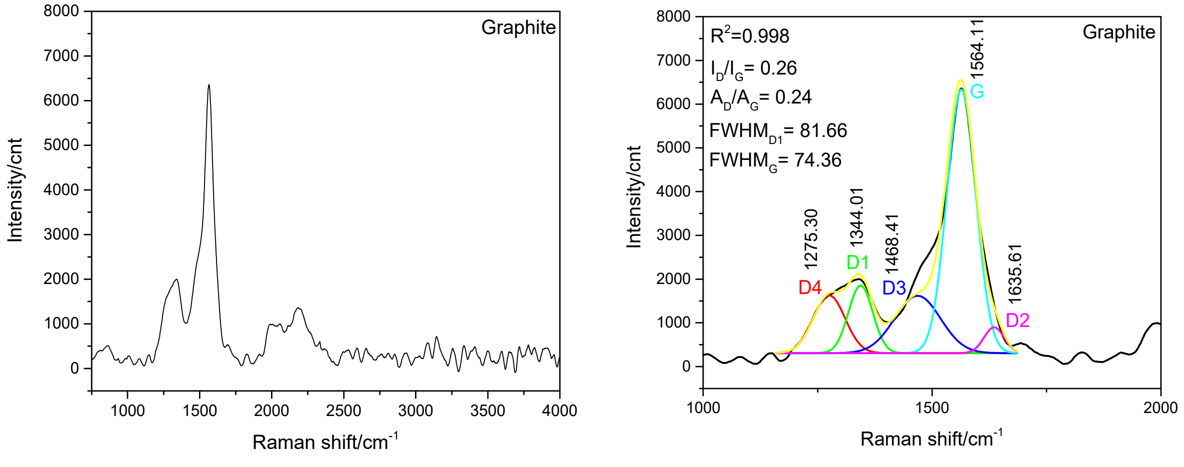

(a) (b)

Figure S6. Raman spectra of graphite (**a**) and the deconvolution of the peaks from the 1st order Raman region of the signal emitted by graphite (**b**). The sum of the deconvolution is marked with yellow line.

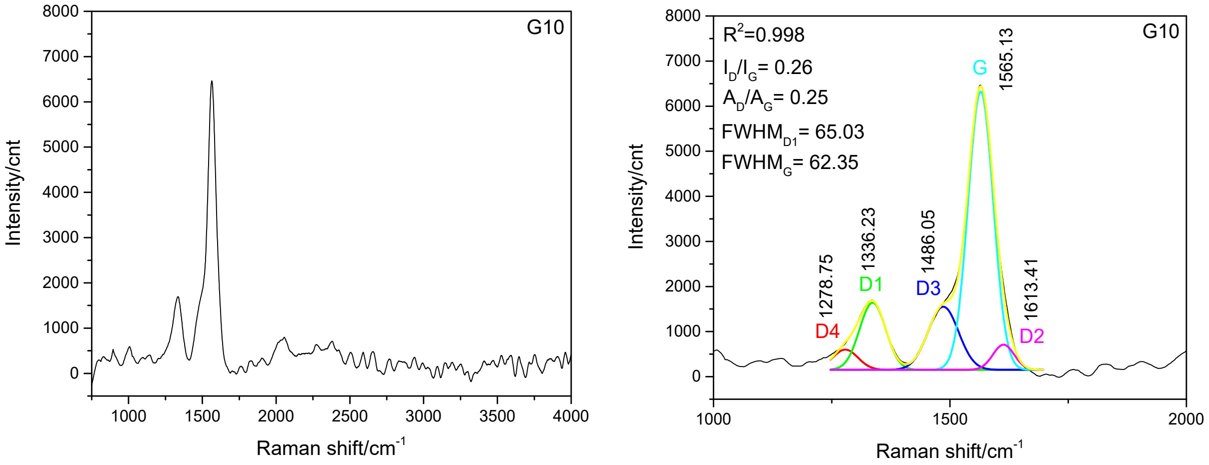

(a) (b)

Figure S7. Raman spectra of G10 sample (**a**) and the deconvolution of the peaks from the 1st order Raman region of the signal emitted by G10 sample (**b**). The sum of the deconvolution is marked with yellow line.

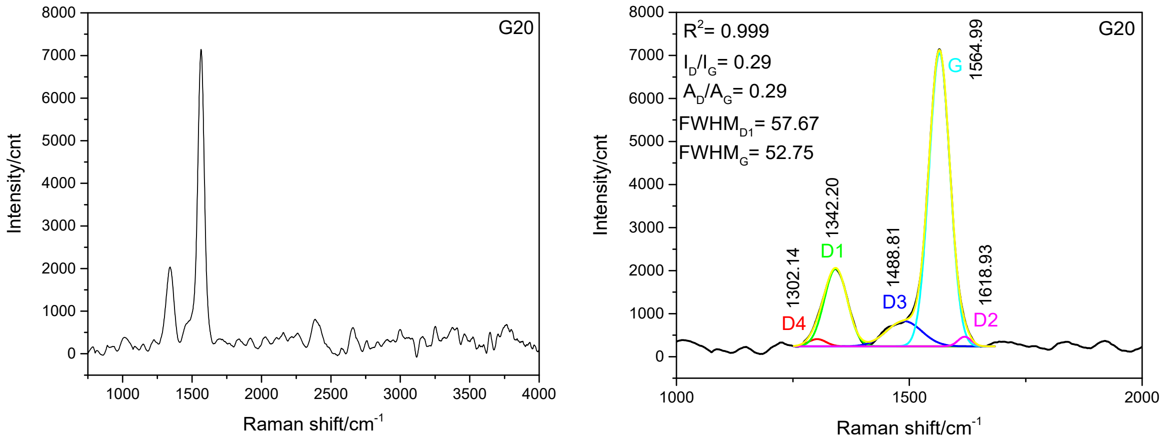

(a) (b)

Figure S8. Raman spectra of G20 sample (**a**) and the deconvolution of the peaks from the 1st order Raman region of the signal emitted by G20 sample (**b**). The sum of the deconvolution is marked with yellow line.

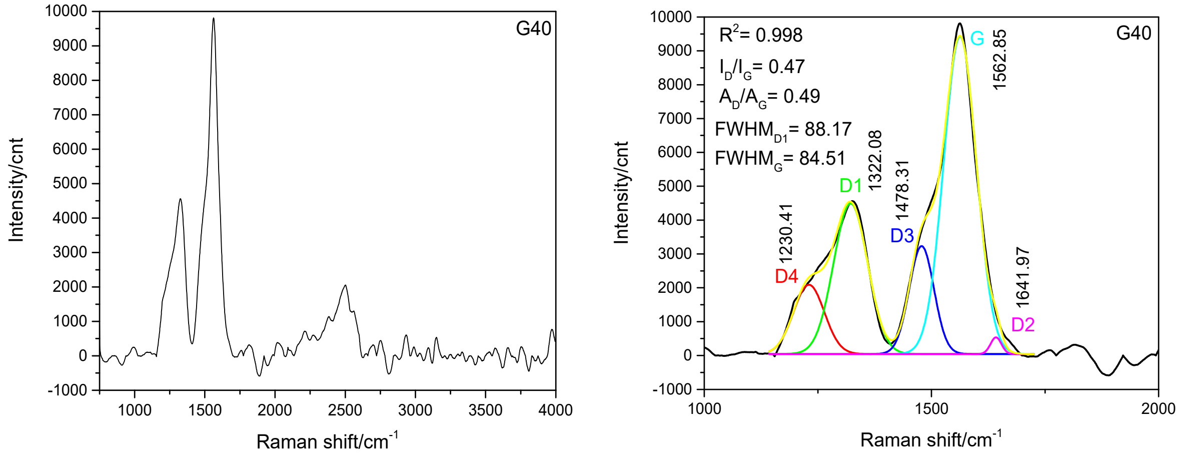

(a) (b)

Figure S9. Raman spectra of G40 sample (**a**) and the deconvolution of the peaks from the 1st order Raman region of the signal emitted by G40 sample (**b**). The sum of the deconvolution is marked with yellow line.

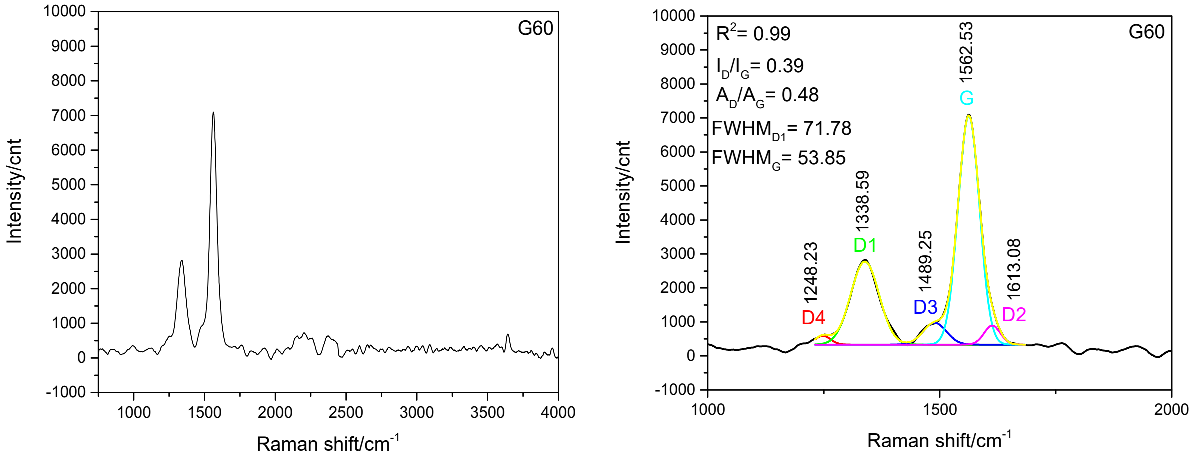

(a) (b)

Figure S10. Raman spectra of G60 sample (**a**) and the deconvolution of the peaks from the 1st order Raman region of the signal emitted by G60 sample (**b**). The sum of the deconvolution is marked with yellow line.

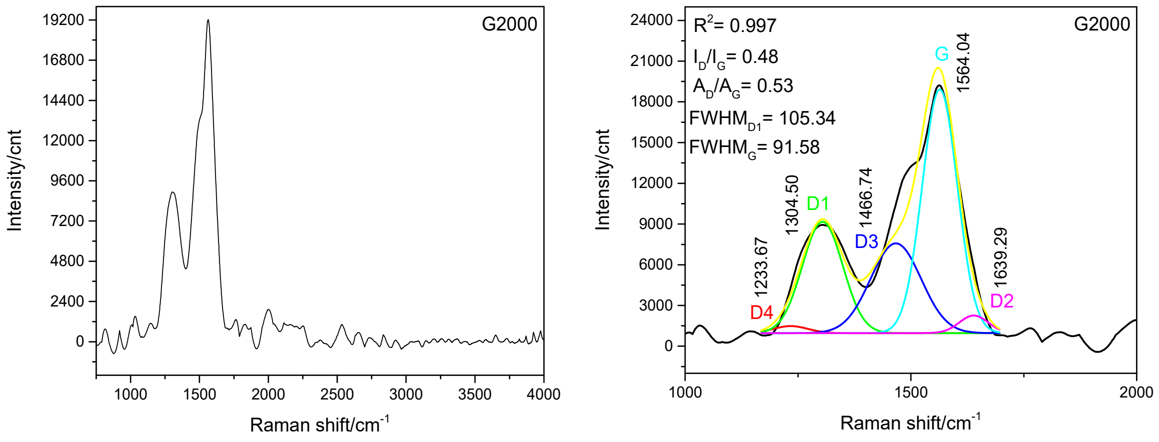

(a) (b)

Figure S11. Raman spectra of G@2000 sample (**a**) and the deconvolution of the peaks from the 1st order Raman region of the signal emitted by G@2000 sample (**b**). The sum of the deconvolution is marked with yellow line.

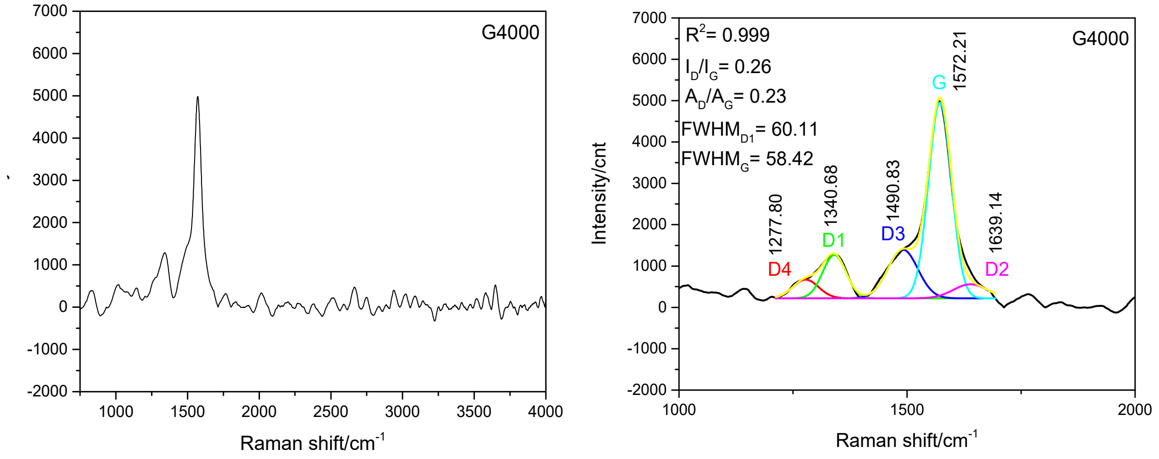

(a) (b)

Figure S12. Raman spectra of G@4000 sample (**a**) and the deconvolution of the peaks from the 1st order Raman region of the signal emitted by G@4000 sample (**b**). The sum of the deconvolution is marked with yellow line.

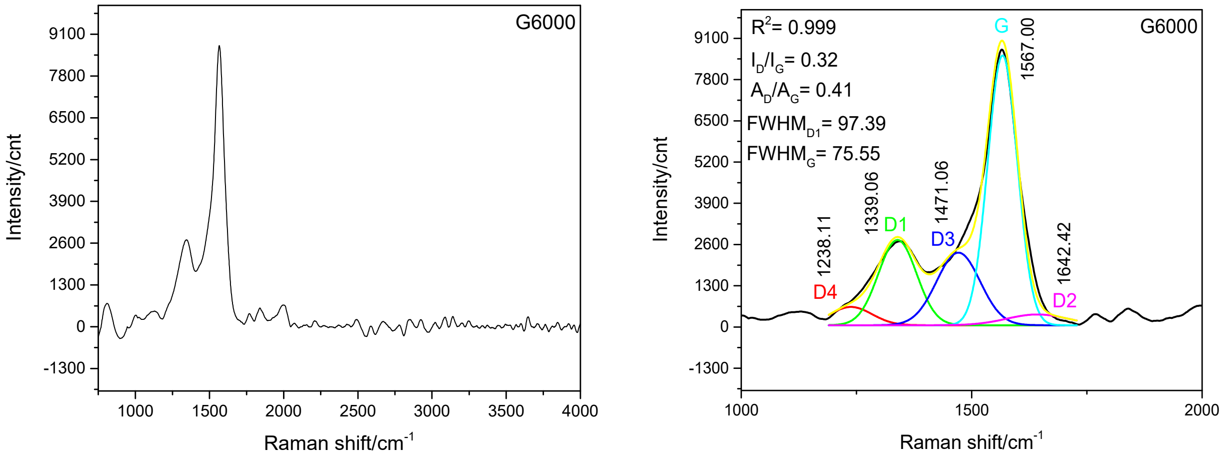

(a) (b)

Figure S13. Raman spectra of G@6000 sample (**a**) and the deconvolution of the peaks from the 1st order Raman region of the signal emitted by G@6000 sample (**b**). The sum of the deconvolution is marked with yellow line.

Table S1. Raman data related to the G and D bands locations and ID/IG ratio for all the studied carbon materials.

	**G band/ cm $^{-1}$**	**D band/ cm $^{-1}$**	**I_D/ I_G**
**G_REF**	1564.56 ± 0.18	1380.47 ± 0.81	0.15
**Graphite**	1564.10 ± 0.10	1344.00 ± 3.63	0.26
**G10**	1565.13 ± 0.73	1336.67 ± 2.41	0.26
**G20**	1565.00 ± 0.06	1342.20 ± 1.13	0.29
**G40**	1562.85 ± 0.29	1322.08 ± 0.79	0.48
**G60**	1562.54 ± 0.09	1338.59 ± 0.11	0.39
**G@2000**	1564.06 ± 3.76	1304.50 ± 4.51	0.48
**G@4000**	1572.21 ± 0.42	1340.69 ± 2.17	0.26
**G@6000**	1567.00 ± 0.71	1339.06 ± 3.33	0.32

Electrochemical studies: IKA ULTRA-TURRAX® Tube Drive equipment

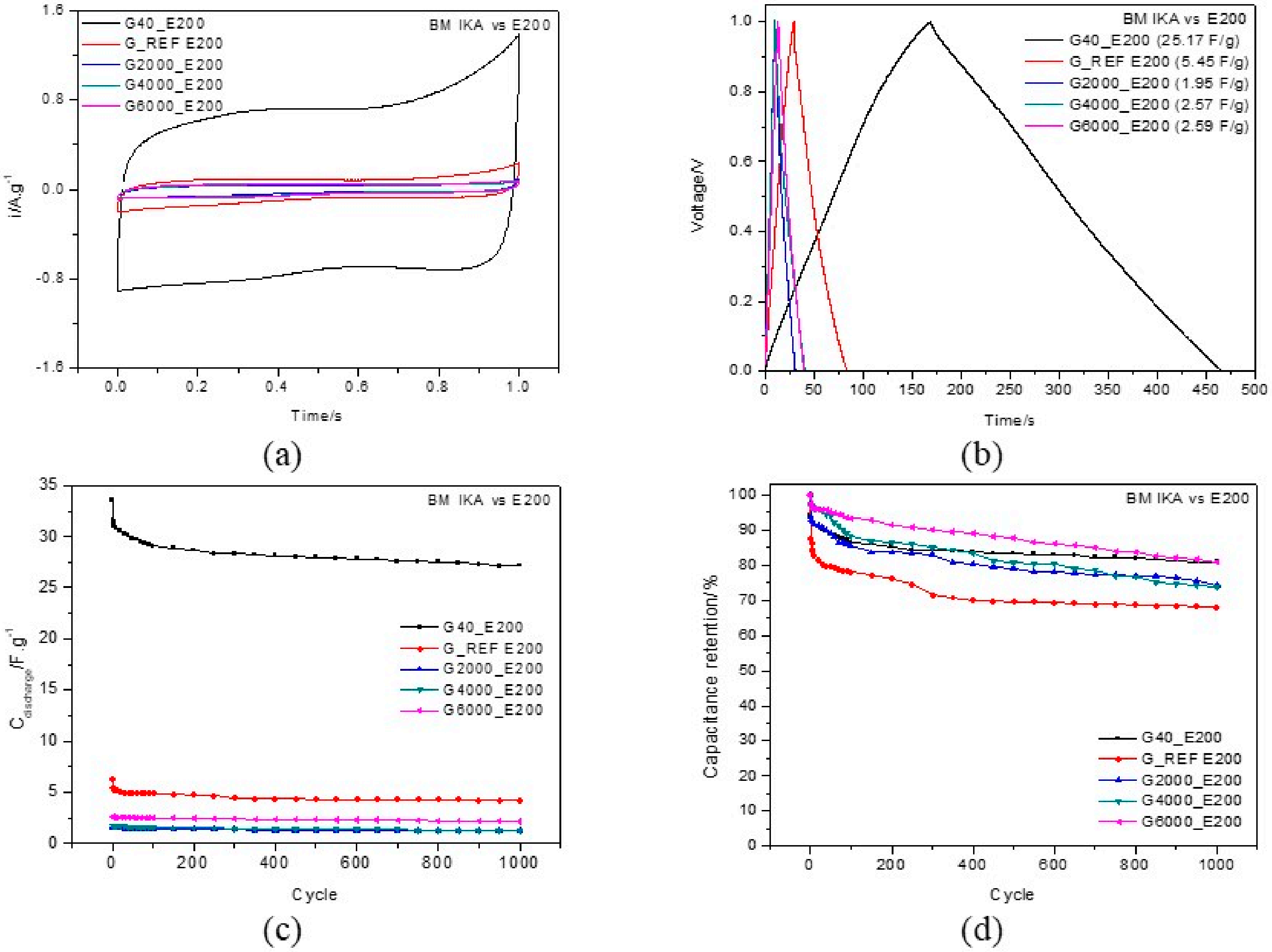

Figure S14. Ball milling effect using IKA ULTRA-TURRAX® Tube Drive for G_REF, G@2000, G@4000 and G@6000 at 30 °C, with comparison with G40 sample. (**a**) cyclic voltammetry; (**b**) galvanostatic charge-discharge curves recorded with current density 1A.g^{-1}; (**c**) discharge gravimetric capacitance for 1000 cycles; (**d**) capacitance retention.

Temperature effect on capacitance

Table S2. Temperature effect on capacitance for the different ball-milled carbon materials.

		1st Cycle				1000th Cycle			
		30°C	40°C	50°C	60°C	30°C	40°C	50°C	60°C
		Capacitance (F/g)				Capacitance (F/g)			
	**G_REF**	**5.45**	**6.85**	**6.96**	**9.11**	**4.35**	**4.53**	5.16	5.9
	**Graphite**	4.27	4.33	4.51	5.09	1.71	1.82	2.03	2.27
	**G10**	2.36	2.88	3.43	4.09	2.11	2.61	3.1	3.8
	**G20**	24.83	25.33	25.98	26.74	19.8	22.31	23.58	25.01
**E200**	**G40**	25.1	25.89	26.47	27.88	23.1	24.87	25.41	26.09
	**G60**	4.19	5.01	5.54	5.96	3.99	4.31	4.92	5.33
	**G@2000**	1.95	2.8	3.89	5.01	1.28	2.41	3.07	4.38
	**G@4000**	2.57	3.77	4.61	5.33	1.39	3.09	4.03	4.87
	**G@6000**	2.59	3.91	4.84	5.55	2.35	3.64	4.31	4.97

Extra studies: IKA ULTRA-TURRAX® Tube Drive equipment

Several experiments were performed with the ULTRA-TURRAX, changing the time and the rotation rate as can be verified in the figure and table below. 1 h of treatment gave the better results, although only residual changes in the specific capacitance can be observed. For times longer than 1 h, ULTRA-TURRAX plastic vessels revealed signs of strong material wear and in some cases even the rupture of the vessel was observed. The capacitance retention after 1000 charge-discharge cycles is of 83.4% for all these conditions.

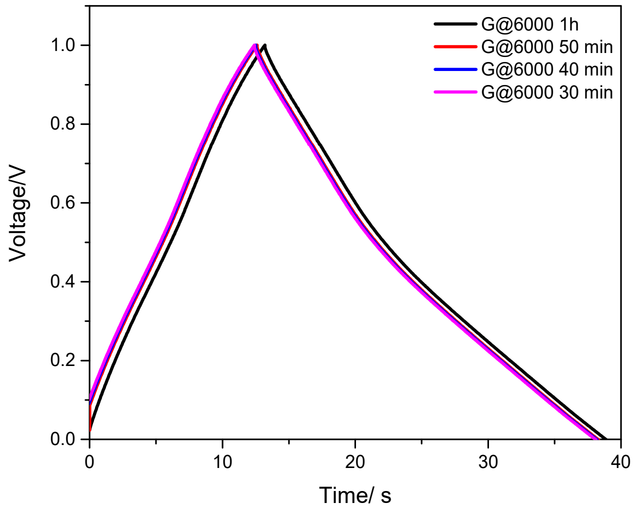

(a)

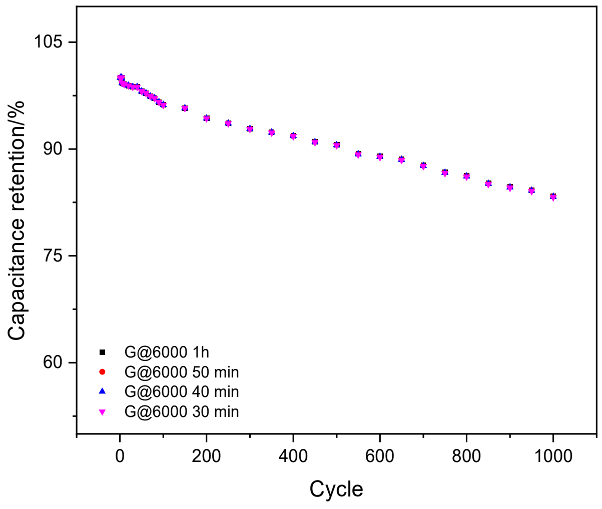

(b)

Figure S15. Ball milling time effect using IKA ULTRA-TURRAX® Tube Drive for G_REF G@6000 at 30 °C. (a) galvanostatic charge-discharge curves recorded with current density 1A.g^{-1}; (b) capacitance retention for 1000 cycles.

Table S3. specific capacitance determined for IKA ULTRA-TURRAX® Tube Drive G@6000 with different milling time (30, 40, 50 and 60 minutes).

Samples	Specific Capacitance (F. g^{-1})
**G@6000 _ 1h**	2.59 ±0.31
**G@6000 _ 50 min**	2.58 ±0.28
**G@6000 _ 40 min**	2.58 ±0.17
**G@6000 _ 30 min**	2.57 ±0.33
